# Explainable Artificial Intelligence Assisted Modeling of Malachite Green Adsorption onto SBA-15–Zn–Fe Composite

**DOI:** 10.3390/molecules31173005

**Published:** 2026-08-27

**Authors:** Memduha Ergüt, Salih Ozbay, Seda Karateke, Metin Zontul

**Affiliations:** 1 Department of Chemical Engineering, Faculty of Engineering and Natural Sciences, Sivas University of Science and Technology, 58000 Sivas, Türkiye; memduha.ergut@sivas.edu.tr (M.E.); salihozbay@sivas.edu.tr (S.O.); 2 Department of Software Engineering, Faculty of Engineering and Natural Sciences, Istanbul Atlas University, 34408 Istanbul, Türkiye; 3 Department of Computer Engineering, Faculty of Engineering and Natural Sciences, Sivas University of Science and Technology, 58000 Sivas, Türkiye; metinzontul@sivas.edu.tr

**Keywords:** malachite green adsorption, SBA-15–Zn–Fe composite, multilayer perceptron (MLP), explainable artificial intelligence (XAI), SHAP analysis, data leakage

## Abstract

Dye-containing industrial effluents pose substantial risks to aquatic ecosystems. In this study, a material prepared using the stated SBA-15–Zn–Fe synthesis procedure was evaluated for malachite green (MG) removal and modeled using machine-learning and explainable artificial intelligence techniques. The dataset comprised 315 experimental observations covering initial MG concentrations of 100–500 mg L^−1^, adsorbent concentrations of 0.5–3.0 g L^−1^, pH values of 5–9, temperatures of 25–45 °C, and contact times of 0–360 min. At an initial MG concentration of 500 mg L^−1^, an adsorbent concentration of 1.0 g L^−1^, pH 9.0, 45 °C, and 90 min, the material achieved 99.65% MG removal. The Langmuir-estimated maximum monolayer capacity was 1428.57 mg g^−1^. A multilayer perceptron (MLP) with a 100–50–25–12 hidden-layer architecture, hyperbolic tangent activation, and L-BFGS optimization was developed to predict residual MG concentration. The prespecified MLP achieved an $R^{2}$ of 0.9624 on the strictly held-out 20% internal test subset. Five-fold cross-validation yielded a mean $R^{2}$ of 0.8420 with a fold-wise standard deviation of 0.1273, while pooled contact-time-based LOGO-CV yielded an $R^{2}$ of 0.7411, indicating reduced transferability when entire contact-time groups were excluded from fitting. Nominal 95% CV+ prediction intervals achieved 98.41% empirical coverage on the held-out test subset, although their relatively broad widths indicated non-negligible predictive uncertainty. PFI and SHAP showed that the fitted MLP relied most strongly on contact time and initial MG concentration. Longer contact times were generally associated with lower predicted residual concentrations, whereas higher initial concentrations were associated with higher predicted residual concentrations. Overall, the framework provided an interpretable assessment of MG adsorption within the investigated experimental domain; external validity and extrapolation beyond this domain were not established.

## 1. Introduction

Water pollution has become a major environmental concern because of rapid industrialization, population growth, and the uncontrolled discharge of wastes originating from industrial, agricultural, and domestic activities. These discharges can alter the physicochemical composition of natural water resources and produce adverse effects on aquatic ecosystems and human health [1]. Among the major contaminants found in industrial effluents, heavy metals and synthetic dyes may present substantial environmental and toxicological risks even at relatively low concentrations. Therefore, the development of effective water-treatment technologies remains an important research priority.

Synthetic dyes used in textile and related industries may enter aquatic environments during manufacturing, processing, and wastewater discharge [2,3]. Malachite green (MG) is a cationic triphenylmethane dye that has been widely used in the paper, leather, plastics, textile, and aquaculture industries. Its release into water bodies can reduce light penetration, inhibit photosynthetic activity, and generate toxic, mutagenic, and potentially carcinogenic effects in aquatic organisms [4,5,6,7]. Consequently, the efficient removal of MG from aqueous media is necessary before contaminated effluents are discharged into receiving environments.

Adsorption is widely used for contaminant removal because of its operational flexibility, relatively simple design, and high removal efficiency [8]. Among the adsorbent materials developed for this purpose, SBA-15 has attracted considerable attention because of its high specific surface area, ordered mesoporous structure, narrow pore-size distribution, thermal stability, and ease of surface modification [9,10,11]. Metal-modified SBA-15 materials, including Fe/SBA-15-based composites, have also been investigated to improve the surface reactivity, adsorption performance, and physicochemical stability of the parent mesoporous silica [12].

Several studies have examined SBA-15 and its modified derivatives for the adsorption of organic dyes. Ebrahimpour et al. investigated the simultaneous adsorption of crystal violet, MG, and methylene blue using SBA-15 mesoporous nanoparticles [13], whereas Chaudhuri et al. evaluated SBA-15 for the removal of neutral red, safranin O, reactive red 2, and Congo red [14]. Different modification strategies have subsequently been proposed to enhance the adsorption properties of SBA-15, including magnetic nanocomposite formation [15], metal oxide incorporation [16], amino functionalization [17], polymer grafting [18], biopolymer modification [19], and metal–organic-framework-based composite fabrication [20]. These studies demonstrate that the adsorption properties of SBA-15 can be tailored by modifying its surface chemistry and composite structure.

Despite these advances, the quantitative description of adsorption systems remains challenging because adsorption behavior may be governed simultaneously by equilibrium, kinetic, mass-transfer, and physicochemical effects. Conventional regression and mechanistic models may not adequately capture complex nonlinear interactions among operational variables. Accordingly, data-driven machine-learning methods, particularly artificial neural networks (ANNs), have been increasingly employed to model, predict, and optimize adsorption processes. Elngar et al. reviewed artificial-intelligence applications in adsorption-based wastewater treatment and emphasized their potential to improve predictive accuracy, operational efficiency, and process optimization [21]. El Mallahi et al. and Reynel-Ávila et al. similarly highlighted the growing role of machine-learning approaches in adsorption modeling and optimization [22,23]. Recent reviews have also summarized current developments, methodological limitations, and future research directions for machine-learning-assisted dye-removal processes [24,25].

Artificial intelligence methods have also been applied specifically to MG adsorption. Nascimento et al. developed ANN-based models for MG adsorption onto baru fruit endocarp [26], while Karishma et al. modeled adsorption behavior using raw and carbonized *Spirogyra maxima* biomass [27]. Sudarsan et al. reported that ANN and adaptive neuro-fuzzy inference system (ANFIS)-based approaches outperformed conventional regression methods for MG adsorption onto superparamagnetic activated carbon and identified contact time as an influential process variable [28]. In a related study, the same research group combined experimental analysis with ANN and ANFIS models to investigate MG adsorption onto magnetic activated carbon [29]. Collectively, these studies indicate that nonlinear predictive models can provide useful representations of MG adsorption behavior when the experimental design includes multiple interacting process variables.

The predictive performance of ANN models depends strongly on network architecture, activation functions, optimization algorithms, data preprocessing, and validation design. Tayebi et al. compared multilayer perceptron (MLP), radial basis function, and group method of data handling models for dye adsorption onto an SBA-15-based hybrid adsorbent and reported favorable predictive performance for the MLP approach [30]. Wang et al. evaluated different optimization algorithms, activation functions, and hidden-neuron configurations for ANN-based MG adsorption modeling and demonstrated the importance of appropriate hyperparameter selection [31]. Alsaffar et al. also showed that activation-function choice and hidden-layer size can substantially affect ANN performance in adsorption-capacity prediction [32].

However, high apparent prediction accuracy does not necessarily imply that a model will generalize reliably to experimentally distinct conditions. Randomly partitioning observations that share common experimental structures may allow closely related samples to appear in both the training and validation subsets, thereby producing overoptimistic performance estimates through data leakage [33]. Furthermore, machine-learning models often exhibit reduced performance when applied to conditions or material domains that are not adequately represented during training [34]. These limitations indicate that conventional randomly partitioned cross-validation should be complemented by validation strategies that preserve relevant experimental group structures.

A further limitation of high-performing ANN and MLP models is their black-box character, which restricts direct interpretation of how the input variables contribute to individual predictions. Explainable artificial intelligence (XAI) methods can partially address this limitation by quantifying global feature importance and local feature-specific contributions. Khajavian et al. reviewed the application of SHapley Additive exPlanations (SHAP) in adsorption-based environmental remediation and emphasized its potential to improve the transparency and interpretability of machine-learning models [35].

In this study, MG adsorption onto an SBA-15–Zn–Fe composite was experimentally investigated and modeled using an MLP trained with the hyperbolic tangent activation function and the limited-memory Broyden–Fletcher–Goldfarb–Shanno algorithm. In addition to conventional five-fold cross-validation, contact-time-based leave-one-group-out cross-validation was employed to evaluate the transferability of the model to contact-time groups excluded from the corresponding training iterations. Permutation Feature Importance and SHAP analyses were subsequently applied to quantify the global and local contributions of the operational variables to the predicted residual MG concentration.

Although MLP models and XAI methods have increasingly been applied to adsorption-process modeling, their combined use alone does not constitute methodological novelty. Many existing studies rely primarily on randomly partitioned validation data and a single post hoc explanation method, which may produce optimistic performance estimates when structurally related observations are distributed across the training and validation subsets and may not distinguish global model reliance from local directional effects. The distinctive contribution of the present study is therefore the integration of three complementary elements within a unified framework: (i) nonlinear MLP-based prediction of residual MG concentration for the experimentally investigated SBA-15–Zn–Fe adsorption system; (ii) contact-time-based leave-one-group-out cross-validation, with preprocessing conducted independently within each training partition, to reduce group-related information leakage and assess transferability to contact-time conditions excluded from model fitting; (iii) complementary PFI and SHAP analyses that quantify, respectively, global predictive reliance and local directional feature contributions. This framework enables predictive accuracy, robustness to grouped experimental conditions, and model-based physicochemical interpretation to be evaluated jointly. Thus, the scientific contribution lies not in the individual application of MLP, PFI, or SHAP, but in their group-aware and complementary integration for the transparent assessment of the SBA-15–Zn–Fe/MG adsorption system within the investigated experimental domain.

## 2. Materials and Methods

### 2.1. Materials

All chemicals were of analytical grade and used without further purification. Tetraethyl orthosilicate (TEOS), Pluronic P123, cetyltrimethylammonium bromide (CTAB), ${\mathrm{FeSO}}_{4}\cdot 7H_{2}$O, zinc powder, polyvinylpyrrolidone (PVP, $M_{w}\approx 55,000$), HCl, H_2_SO_4_, ethanol, and NaOH were obtained from Sigma-Aldrich (St. Louis, MO, USA) or Merck (Darmstadt, Germany). Malachite Green oxalate (C.I. 42,000, $\lambda_{\max}=618$ nm, $M_{w}=365$ g/mol) was obtained from Sigma-Aldrich and used as the cationic dye.

### 2.2. Synthesis of the SBA-15–Zn–Fe Composite

SBA-15 was synthesized by using triblock copolymer P123 (${PEO}_{20}{\mathrm{PPO}}_{70}{\mathrm{PEO}}_{20}$). Briefly, 3 g of P123 was dissolved in 60 mL of HCl (1.5M). Then, 0.6 g of CTAB was dissolved in 25 mL of pure water and added to the P123 solution together with 20 mL of ethanol to prepare the surfactant solution. After mixing at room temperature, 10 mL of TEOS was added dropwise, and the mixture was stirred at 35 °C for 5 h, and then the obtained mixture was kept in an oven under static conditions at 90 °C for 3 days. The resulting solid material was filtered, washed with pure water, and calcined at 540 °C for 5 h to obtain SBA-15 [36,37].

For the synthesis of the SBA-15–Zn–Fe composite, 1.5 g of zinc powder and 0.5 g of SBA-15 were mixed, followed by the addition of 2 mL of 1% (w/v) PVP solution. The mixture was stirred for 30 min, dried at 50 °C for 12 h, and then calcined at 550 °C for 2 h to obtain the SBA-15–Zn composite. After that, 0.5 g of ${\mathrm{FeSO}}_{4}\cdot 7H_{2}$O was dissolved in 200 mL of distilled water adjusted to pH 3.0 by using H_2_SO_4_, and 1 g of SBA-15–Zn was added to the solution. The mixture was stirred for 1 h at room temperature, and the obtained SBA-15–Zn–Fe composite was separated by centrifugation (5000 rpm, 5 min), washed with deionized water, and dried at 105 °C. PVP was used as a binding agent to promote the association of zinc particles with SBA-15. Subsequently, the SBA-15–Zn material was treated with ${\mathrm{FeSO}}_{4}$ under conditions intended to promote a chemical replacement reaction between ${\mathrm{Fe}}^{2+}$ and Zn^0^, following the procedure reported in [38]. Because the elemental composition and chemical states of the metal-containing species were not independently characterized, the occurrence and extent of this proposed replacement reaction could not be conclusively verified.

The textural parameters of SBA-15 and the SBA-15–Zn–Fe composite were obtained from nitrogen adsorption measurements at 76.256K using a Micromeritics ASAP 2020 (Micromeritics Instrument Corporation, Norcross, GA, USA) surface area and porosity analyzer operated with software version 3.00 G. The specific surface area, $S_{\mathrm{BET}}$, was determined using the Brunauer–Emmett–Teller method, and the average pore diameter, $d_{\mathrm{BJH}}$, was obtained using the Barrett–Joyner–Halenda method. The total pore volume, $V_{T}$, was also obtained from the nitrogen adsorption analysis. The resulting textural parameters are presented in Supplementary Table S2.

### 2.3. Adsorption Experiments

A stock MG solution with a concentration of 1.0 g L^−1^ was prepared by dissolving 1.0 g of MG in 1 L of distilled water. Working solutions with initial MG concentrations ranging from 100 to 500 mg L^−1^ were prepared by diluting the stock solution with distilled water. The initial pH of each solution was adjusted using 0.1 N HCl or 0.1 N NaOH.

Batch adsorption experiments were conducted in 100 mL Erlenmeyer flasks containing 50 mL of MG solution. Except for the experiments specifically designed to investigate the effect of adsorbent concentration, 0.05 g of the SBA-15–Zn–Fe material was added to each flask, corresponding to an adsorbent concentration of 1.0 g L^−1^. The flasks were agitated under constant shaking and the temperature conditions specified in the experimental design. Samples were collected before the addition of the adsorbent and at predetermined contact times during the adsorption experiments. After centrifugation, the residual MG concentration in the supernatant was determined using a Microplate spectrophotometer (Infinite M Nano, TECAN, Grödig (Salzburg), Austria [Tecan Austria GmbH]) at an ultraviolet-visible (UV–vis) wavelength of 618 nm.

The resulting dataset comprised 315 experimental observations. The five input variables were the initial MG concentration ($C_{0}$, 100–500 mg L^−1^), adsorbent concentration ($X_{0}$, 0.5–3.0 g L^−1^), contact time (*t*, 0–360 min), initial solution pH (5–9), and temperature (*T*, 25–45 °C). The residual MG concentration in the liquid phase at contact time *t*, denoted by $C_{t}$ (mg L^−1^), was used as the response variable for machine-learning modeling.

The adsorption capacity at contact time *t*, $q_{t}$ (mg g^−1^), and the equilibrium removal efficiency were calculated as

(1) $$q_{t}=\frac{(C_{0}-C_{t})V}{m}$$

and

(2) $$\mathrm{Removal}\;\mathrm{efficiency}\;\left(\%\right)=100\left(\frac{C_{0}-C_{e}}{C_{0}}\right),$$

respectively, where $C_{0}$, $C_{t}$, and $C_{e}$ (mg L^−1^) denote the initial, time-dependent, and equilibrium MG concentrations, respectively; *V* (L) is the solution volume; and *m* (g) is the adsorbent mass. The adsorbent concentration was defined as $X_{0}=m/V$.

### 2.4. Exploratory Statistical Analysis and Linear Modeling

Statistical analyses were conducted to characterize the experimental dataset and to examine the linear relationships between the operational variables and the residual malachite green (MG) concentration. The independent variables were adsorbent concentration ($X_{0}$, $g\;L^{-1}$), temperature (*T*, °C), initial MG concentration ($C_{0}$, $\mathrm{mg}\;L^{-1}$), initial solution pH, and contact time (*t*, min). The residual MG concentration at contact time *t*, denoted by $C_{t}$ ($\mathrm{mg}\;L^{-1}$), was treated as the dependent variable.

Descriptive statistics, including the arithmetic mean, standard deviation, minimum, and maximum, were calculated for each variable. These statistics were used to summarize the central tendency, dispersion, and experimental ranges represented in the dataset.

Pairwise linear associations among the experimental variables were evaluated using Pearson’s product–moment correlation coefficient [39]. For two variables *X* and *Y*, the Pearson correlation coefficient was calculated as

(3) $$r_{XY}=\frac{\sum_{i=1}^{N}\left(X_{i}-\bar{X}\right)\left(Y_{i}-\bar{Y}\right)}{\sqrt{\sum_{i=1}^{N}{\left(X_{i}-\bar{X}\right)}^{2}}\sqrt{\sum_{i=1}^{N}{\left(Y_{i}-\bar{Y}\right)}^{2}}}.$$

In Equation (3), N=315 is the number of experimental observations, $X_{i}$ and $Y_{i}$ are the observed values of the two variables, and $\bar{X}$ and $\bar{Y}$ are their corresponding sample means. A correlation matrix was constructed to evaluate the direction and strength of the pairwise linear associations among $X_{0}$, *T*, $C_{0}$, pH, *t*, and $C_{t}$. The resulting coefficients were interpreted as measures of linear association rather than evidence of causal relationships [39].

A multiple linear regression (MLR) model was subsequently fitted using the ordinary least squares (OLS) method to quantify the simultaneous linear relationships between the five operational variables and the residual MG concentration [40]. The regression model was specified as

(4) $$C_{t,i}=\beta_{0}+\beta_{1}X_{0,i}+\beta_{2}T_{i}+\beta_{3}C_{0,i}+\beta_{4}{\mathrm{pH}}_{i}+\beta_{5}t_{i}+\varepsilon_{i},\;i=1,\ldots ,N,$$

where $\beta_{0}$ is the intercept, $\beta_{1},\ldots ,\beta_{5}$ are the regression coefficients, and $\varepsilon_{i}$ denotes the residual error associated with the *i*-th observation.

The OLS estimate of the coefficient vector was obtained by minimizing the residual sum of squares:

(5) $$\hat{\beta }=\underset{\beta }{\mathrm{arg min}}\sum_{i=1}^{N}{\left(C_{t,i}-{\hat{C}}_{t,i}\right)}^{2},$$

where ${\hat{C}}_{t,i}$ is the residual MG concentration predicted by the fitted linear regression model.

The proportion of the observed variability in $C_{t}$ explained by the MLR model was evaluated using the coefficient of determination:

(6) $$R^{2}=1-\frac{\sum_{i=1}^{N}{\left(C_{t,i}-{\hat{C}}_{t,i}\right)}^{2}}{\sum_{i=1}^{N}{\left(C_{t,i}-{\bar{C}}_{t}\right)}^{2}},$$

where ${\bar{C}}_{t}$ denotes the sample mean of the measured residual MG concentrations.

The statistical significance of each regression coefficient was evaluated using its corresponding *t*-statistic and two-sided *p*-value. Analysis of variance (ANOVA) was used to partition the variability associated with the fitted regression model and the unexplained residual error, and to calculate the corresponding predictor-level *F*-statistics [40]. A nominal significance level of $\alpha_{\mathrm{sig}}=0.05$ was adopted. Accordingly, $p<\alpha_{\mathrm{sig}}$ was considered statistically significant. Nevertheless, the *p*-values were interpreted together with the signs and magnitudes of the estimated coefficients and were not treated as independent measures of scientific or practical importance [41].

The linear analyses were used as an exploratory benchmark rather than as the principal predictive model. In particular, the MLR results provided a reference for assessing whether nonlinear modeling was warranted and for comparing the statistical associations with the feature-importance patterns subsequently obtained from the MLP, PFI, and SHAP analyses.

All statistical calculations and graphical analyses were performed in Python 3.13. The OLS regression and ANOVA procedures were implemented using the statsmodels statistical modeling library [42].

### 2.5. Multilayer Perceptron (MLP) Modeling

An MLP is a feedforward artificial neural network capable of representing complex nonlinear relationships between input and output variables [43]. In this study, the MLP consisted of an input layer with five nodes, four hidden layers containing 100, 50, 25, and 12 neurons, respectively, and a single-node regression output layer. The specific layer widths were prespecified as an expansion–tapering architecture rather than identified through an exhaustive architecture or hyperparameter search.

The first hidden layer maps the five standardized physicochemical input variables into an expanded nonlinear representation, thereby providing sufficient representational capacity for modeling interactions among the adsorption parameters. The subsequent reduction in hidden-layer width from 100 to 50, 25, and 12 neurons progressively produces a more compact internal representation before the scalar output. This structural rationale explains the intended role of the architecture but does not establish that the selected configuration is globally optimal, physically constrained, or inherently resistant to overfitting. Its predictive adequacy was therefore evaluated empirically using the validation procedures described in Section 2.6.

For the *i*-th experimental observation, the input vector was defined as

(7) $$x_{i}={\left[\begin{array}{ccccc} X_{0,i} & T_{i} & C_{0,i} & {\mathrm{pH}}_{i} & t_{i} \end{array}\right]}^{T}\in R^{5}.$$

Here, $X_{0}$ denotes the adsorbent concentration, *T* is the temperature, $C_{0}$ is the initial MG concentration, pH is the initial solution pH, and *t* is the contact time. The response variable was the residual MG concentration $C_{t,i}$.

Because the input variables were measured in different physical units and numerical ranges, each feature was standardized before model training [44]. For the *j*-th feature of the *i*-th observation, the standardized value was calculated as

(8) $${\tilde{x}}_{ij}=\frac{x_{ij}-\mu_{j,\mathrm{tr}}}{\sigma_{j,\mathrm{tr}}},\;j=1,\ldots ,5.$$

where $\mu_{j,\mathrm{tr}}$ and $\sigma_{j,\mathrm{tr}}$ are the mean and standard deviation of the *j*-th feature calculated exclusively from the corresponding training subset.

The response variable was also standardized using statistics calculated exclusively from the corresponding training subset:

(9) $${\tilde{C}}_{t,i}=\frac{C_{t,i}-\mu_{C_{t},\mathrm{tr}}}{\sigma_{C_{t},\mathrm{tr}}}.$$

In Equation (9), $\mu_{C_{t},\mathrm{tr}}$ and $\sigma_{C_{t},\mathrm{tr}}$ denote the training-subset mean and standard deviation of the residual MG concentration. The same training-derived transformations were applied to the corresponding validation and test observations without re-estimation.

Let ${\tilde{x}}_{i}={\left[\begin{array}{ccccc} {\tilde{x}}_{i1} & {\tilde{x}}_{i2} & {\tilde{x}}_{i3} & {\tilde{x}}_{i4} & {\tilde{x}}_{i5} \end{array}\right]}^{T}$ denote the standardized input vector. The output of the input layer was defined as

(10) $$h_{i}^{\left(0\right)}={\tilde{x}}_{i}.$$

For the *ℓ*-th hidden layer, the affine transformation and nonlinear activation operations were defined as

(11) $$z_{i}^{\left(\ell \right)}=W^{\left(\ell \right)}h_{i}^{\left(\ell -1\right)}+b^{\left(\ell \right)},\;h_{i}^{\left(\ell \right)}=f\;\left(z_{i}^{\left(\ell \right)}\right),\;\ell =1,2,3,4,$$

where $W^{\left(\ell \right)}$ is the weight matrix, $b^{\left(\ell \right)}$ is the bias vector, $z_{i}^{\left(\ell \right)}$ is the pre-activation vector, and $h_{i}^{\left(\ell \right)}$ is the output of the corresponding hidden layer.

The hyperbolic tangent activation function was applied componentwise in all hidden layers and was defined as

(12) $$f\left(z\right)=\tanh\left(z\right)=\frac{\exp(z)-\exp(-z)}{\exp(z)+\exp(-z)}.$$

According to Equations (11) and (12), the outputs of the four hidden layers were obtained successively as

(13) $$\begin{array}{cc} h_{i}^{\left(1\right)}\; & =\tanh\;\left(W^{\left(1\right)}{\tilde{x}}_{i}+b^{\left(1\right)}\right),\\ h_{i}^{\left(2\right)}\; & =\tanh\;\left(W^{\left(2\right)}h_{i}^{\left(1\right)}+b^{\left(2\right)}\right),\\ h_{i}^{\left(3\right)}\; & =\tanh\;\left(W^{\left(3\right)}h_{i}^{\left(2\right)}+b^{\left(3\right)}\right),\\ h_{i}^{\left(4\right)}\; & =\tanh\;\left(W^{\left(4\right)}h_{i}^{\left(3\right)}+b^{\left(4\right)}\right). \end{array}$$

For the selected 100–50–25–12 hidden-layer configuration, the dimensions of the weight matrices and bias vectors were

(14) $$\begin{array}{cccc} W^{\left(1\right)} & \in R^{100\times 5},\; & b^{\left(1\right)} & \in R^{100},\\ W^{\left(2\right)} & \in R^{50\times 100},\; & b^{\left(2\right)} & \in R^{50},\\ W^{\left(3\right)} & \in R^{25\times 50},\; & b^{\left(3\right)} & \in R^{25},\\ W^{\left(4\right)} & \in R^{12\times 25},\; & b^{\left(4\right)} & \in R^{12}. \end{array}$$

Because the response variable was continuous, an identity activation function was used in the single-node output layer. The predicted standardized residual MG concentration was therefore expressed as

(15) $${\hat{\tilde{C}}}_{t,i}=W^{\left(5\right)}h_{i}^{\left(4\right)}+b^{\left(5\right)}.$$

The output-layer weight matrix and bias had the dimensions $W^{\left(5\right)}\in R^{1\times 12},\;b^{\left(5\right)}\in R.$

Predictions on the original residual-concentration scale were obtained using the inverse transformation

(16) $${\hat{C}}_{t,i}=\sigma_{C_{t},\mathrm{tr}}{\hat{\tilde{C}}}_{t,i}+\mu_{C_{t},\mathrm{tr}}.$$

Accordingly, the standardized forward propagation of the MLP model was summarized recursively as

(17) $$\begin{array}{cc} h_{i}^{\left(0\right)}\; & ={\tilde{x}}_{i},\\ h_{i}^{\left(\ell \right)}\; & =\tanh\;\left(W^{\left(\ell \right)}h_{i}^{\left(\ell -1\right)}+b^{\left(\ell \right)}\right),\;\ell =1,2,3,4,\\ {\hat{\tilde{C}}}_{t,i}\; & =W^{\left(5\right)}h_{i}^{\left(4\right)}+b^{\left(5\right)}. \end{array}$$

Let

(18) $$\theta =\left\{W^{\left(1\right)},W^{\left(2\right)},W^{\left(3\right)},W^{\left(4\right)},W^{\left(5\right)},b^{\left(1\right)},b^{\left(2\right)},b^{\left(3\right)},b^{\left(4\right)},b^{\left(5\right)}\right\}$$

denote the complete set of trainable model parameters.

For a training subset containing $N_{\mathrm{tr}}$ observations, the network parameters were estimated by minimizing the regularized squared-error objective function

(19) $$J\left(\theta \right)=\frac{1}{2N_{\mathrm{tr}}}\sum_{i=1}^{N_{\mathrm{tr}}}{\left({\tilde{C}}_{t,i}-{\hat{\tilde{C}}}_{t,i}\right)}^{2}+\frac{\alpha_{L2}}{2N_{\mathrm{tr}}}\sum_{\ell =1}^{5}{∥W^{\left(\ell \right)}∥}_{F}^{2},$$

where $\alpha_{L2}\geq 0$ denotes the L2-regularization parameter and ${∥\cdot ∥}_{F}$ denotes the Frobenius norm. The L2-regularization strength used in all model-fitting iterations was fixed at $\alpha_{L2}=0.0001$.

The optimal model parameters were determined by solving $\theta^{*}={\mathrm{arg min}}_{\theta }J\left(\theta \right)$ using the limited-memory Broyden–Fletcher–Goldfarb–Shanno (L-BFGS) algorithm [45]. L-BFGS is a quasi-Newton optimization method that constructs a limited-memory approximation of the inverse Hessian matrix using information from recent parameter and gradient updates. Consequently, it avoids the explicit construction and storage of the complete Hessian matrix.

The L-BFGS solver was selected because model development was performed using a moderate-sized development subset (n=252) containing five input variables, making deterministic full-batch quasi-Newton optimization computationally feasible. Unlike stochastic mini-batch solvers, L-BFGS calculates each parameter update using the complete fitting partition and therefore avoids variability arising from mini-batch sampling. The MLP was fitted using max_iter=2000 and random_state=42, consistent with the implementation used for the independent model comparison.

This numerical rationale should not be interpreted as evidence that L-BFGS regularizes the network or prevents overfitting. The selected 100–50–25–12 architecture contains 7250 trainable weights and biases, exceeding the total number of experimental observations. Consequently, neither the architecture nor the optimizer alone establishes model adequacy. The susceptibility of the model to overfitting was therefore assessed empirically using $L_{2}$ regularization, training-only standardization, five-fold cross-validation, contact-time-based LOGO-CV, and a strictly held-out internal test subset, as described in Section 2.6.

To reduce the risk of data leakage, the input- and response-standardization parameters were estimated separately within each training partition. The fitted transformations were subsequently applied to the corresponding validation or test observations without re-estimation.

The model was fitted using the standardized response variable. Cross-validation and test-set MSE values were therefore calculated on the standardized response scale, whereas predictions were transformed back to the original $\mathrm{mg}\;L^{-1}$ scale for graphical presentation and concentration-based interpretation.

All preprocessing, model construction, optimization, and prediction procedures were implemented in Python 3.13 using the scikit-learn library [46]. The pandas and NumPy libraries were used for data organization, numerical calculations, and matrix operations.

### 2.6. Model Validation and Performance Metrics

To evaluate the predictive reliability and generalization performance of the developed MLP model, a multistage validation strategy was employed. The complete experimental dataset, D, was initially divided into mutually exclusive training and test subsets according to

(20) $$D=D_{\mathrm{tr}}\;\dot{\cup }\;D_{\mathrm{te}},\;\frac{\left|D_{\mathrm{tr}}\right|}{\left|D\right|}=0.80,\;\frac{\left|D_{\mathrm{te}}\right|}{\left|D\right|}=0.20,$$

where $D_{\mathrm{tr}}$ and $D_{\mathrm{te}}$ denote the training and strictly held-out internal test subsets, respectively. The fixed partition was generated using random_state=42, resulting in 252 training observations and 63 test observations. The test subset was separated before feature and response standardization and model fitting. It was not used to determine model configurations, select hyperparameters, or modify the modeling procedure.

For contextual algorithmic benchmarking, the prespecified MLP was compared with Random Forest regression, standard Gradient Boosting regression, and Support Vector Regression (SVR). All four models were trained using the same $D_{\mathrm{tr}}$ subset and evaluated using the same $D_{\mathrm{te}}$ observations. The MLP configuration was fixed as described in Section 2.5. The alternative models retained their scikit-learn default hyperparameters, except for random_state=42 where supported. No cross-validation-based hyperparameter optimization was conducted as part of this algorithmic comparison.

For the independent test-set comparison, the input and response standardization parameters were estimated exclusively from $D_{\mathrm{tr}}$. The fitted transformations were subsequently applied to $D_{\mathrm{te}}$ without re-estimation. Each candidate model was then fitted using the complete standardized training subset and evaluated once on the standardized test response without further configuration changes or tuning. Thus, no information from $D_{\mathrm{te}}$ entered model fitting or model-configuration decisions. The complete model configurations and independent test-set results are reported in Supplementary Table S1.

The five-fold cross-validation and contact-time-based LOGO-CV procedures were conducted separately to evaluate the stability and time-group transferability of the prespecified MLP. These MLP-specific validation procedures were distinct from the single-split algorithmic comparison reported in Supplementary Table S1.

For the MLP-specific validation analysis, all preprocessing and model-fitting operations were performed within the corresponding cross-validation iteration. The input and response scaling parameters were estimated exclusively from the fitting observations in each iteration and were subsequently applied to the associated validation observations without re-estimation. This procedure reduced the risk of preprocessing-related information leakage between the fitting and validation subsets [46,47].

A five-fold cross-validation procedure was first applied to $D_{\mathrm{tr}}$ to assess the stability of the prespecified MLP under different data partitions [48]. The training observations were divided into five mutually exclusive folds of approximately equal size. In each cross-validation iteration, four folds were used to fit the complete preprocessing and MLP pipeline, whereas the remaining fold was used exclusively for validation. This process was repeated until each fold had served once as the validation subset.

Since observations obtained under the same contact-time condition may share a common experimental structure, a contact-time-based leave-one-group-out cross-validation (LOGO-CV) procedure was also performed. Let $G=\left\{g_{1},g_{2},\ldots ,g_{G}\right\}.$ Denote the set of *G* distinct contact-time groups in the training dataset. For a given group g∈G, the corresponding training and validation subsets were defined as

(21) $$D_{\mathrm{val}}^{\left(g\right)}=\left\{\left(x_{i},y_{i}\right)\in D_{\mathrm{tr}}:t_{i}=g\right\},\;D_{\mathrm{fit}}^{\left(g\right)}=D_{\mathrm{tr}}\setminus D_{\mathrm{val}}^{\left(g\right)}.$$

In each LOGO-CV iteration, all observations associated with one contact time were excluded from model fitting and used together as the validation group. The procedure was repeated *G* times so that each contact-time group was used exactly once for validation. This group-aware validation strategy was employed to assess the ability of the model to predict observations belonging to contact-time conditions that were not included in the corresponding training iteration [49]. Thus, LOGO-CV provided a more restrictive evaluation of time-group transferability than conventional randomly partitioned cross-validation. However, holding out an observed contact-time group does not necessarily constitute extrapolation beyond the overall experimental time range.

The predictive performance of the model was quantified using the mean squared error (MSE) and the coefficient of determination ($R^{2}$). For an evaluation subset containing $n_{e}$ observations, MSE was calculated as

(22) $$\mathrm{MSE}=\frac{1}{n_{e}}\sum_{i=1}^{n_{e}}{\left(y_{i}-{\hat{y}}_{i}\right)}^{2},$$

where $y_{i}=C_{t,i}$ is the experimentally measured residual MG concentration and ${\hat{y}}_{i}={\hat{C}}_{t,i}$ is the corresponding value predicted by the MLP model. Lower MSE values indicate smaller average squared prediction errors. Because MSE is scale-dependent, its numerical value and unit depend on the scale used for the response variable during evaluation.

The coefficient of determination was calculated as

(23) $$R^{2}=1-\frac{\sum_{i=1}^{n_{e}}{\left(y_{i}-{\hat{y}}_{i}\right)}^{2}}{\sum_{i=1}^{n_{e}}{\left(y_{i}-{\bar{y}}_{e}\right)}^{2}},$$

where

(24) $${\bar{y}}_{e}=\frac{1}{n_{e}}\sum_{i=1}^{n_{e}}y_{i}$$

is the mean of the experimentally measured response values within the corresponding evaluation subset. An $R^{2}$ value approaching unity indicates strong agreement between the experimental and predicted values. A value of zero indicates performance equivalent to predicting the evaluation-set mean, whereas a negative value indicates performance inferior to that reference prediction.

For a cross-validation procedure containing *K* folds, the mean value of a performance metric $s_{k}$, where $s_{k}$ represents either the fold-specific MSE or $R^{2}$, was calculated as

(25) $${\bar{s}}_{\mathrm{CV}}=\frac{1}{K}\sum_{k=1}^{K}s_{k}.$$

The variability of the fold-specific scores was summarized using the sample standard deviation:

(26) $$s_{\mathrm{CV}}=\sqrt{\frac{1}{K-1}\sum_{k=1}^{K}{\left(s_{k}-{\bar{s}}_{\mathrm{CV}}\right)}^{2}}.$$

For five-fold cross-validation, K=5. For LOGO-CV, K=G, where *G* is the number of distinct contact-time groups. Accordingly, both the mean and standard deviation of the fold-specific performance values can be reported for LOGO-CV. If the LOGO-CV predictions from all held-out groups are combined and evaluated collectively, the resulting quantities should be identified explicitly as pooled out-of-fold MSE and pooled out-of-fold $R^{2}$, rather than as mean cross-validation scores.

Predictive uncertainty was quantified using five-fold cross-validation-plus (CV+) [50]. This method constructs prediction intervals from out-of-fold residuals without requiring a parametric distributional assumption, such as normality, for the prediction errors. The analysis was conducted exclusively within the development subset $D_{\mathrm{tr}}$, using the same five-fold partitioning strategy described above. The responses in the strictly held-out internal test subset $D_{\mathrm{te}}$ were not used for interval construction or calibration.

For each fold $S_{k}$, the input and response scalers were estimated using only the observations in $D_{\mathrm{tr}}\setminus S_{k}$. The MLP was then fitted on this fitting partition and used to predict both the observations in the excluded fold and the covariates in $D_{\mathrm{te}}$. For each observation $i\in S_{k}$, the out-of-fold absolute residual, calculated after transforming the prediction back to the original concentration scale $\left(\mathrm{mg}\;L^{-1}\right)$, was defined as $r_{i}^{\mathrm{CV}}=\left|y_{i}-{\hat{m}}_{-S_{k}}\left(x_{i}\right)\right|,$ where ${\hat{m}}_{-S_{k}}$ denotes the complete preprocessing and MLP pipeline fitted without fold $S_{k}$.

For each test covariate vector $x_{j}$, the lower and upper CV+ candidates associated with calibration observation *i* were defined as $L_{ij}={\hat{m}}_{-S_{k(i)}}\left(x_{j}\right)-r_{i}^{\mathrm{CV}},\;U_{ij}={\hat{m}}_{-S_{k(i)}}\left(x_{j}\right)+r_{i}^{\mathrm{CV}},$ where k(i) identifies the fold containing observation *i*. The prediction-interval miscoverage level was denoted by $\alpha_{\mathrm{PI}}$ and fixed at $\alpha_{\mathrm{PI}}=0.05$, corresponding to a nominal coverage of $1-\alpha_{\mathrm{PI}}=0.95$.

Let $n_{\mathrm{cal}}=\left|D_{\mathrm{tr}}\right|=252$ denote the number of calibration residuals. The finite-sample lower and upper order-statistic ranks were calculated as

$$\begin{array}{cc} r_{\mathrm{low}} & =\max\left\{1,\left\lfloor \alpha_{\mathrm{PI}}\left(n_{\mathrm{cal}}+1\right)\right\rfloor \right\},\\ r_{\mathrm{up}} & =\min\left\{n_{\mathrm{cal}},\left\lceil \left(1-\alpha_{\mathrm{PI}}\right)\left(n_{\mathrm{cal}}+1\right)\right\rceil \right\}. \end{array}$$

The nominal 95% CV+ prediction interval for $x_{j}$ was subsequently constructed as

$${\hat{C}}_{1-\alpha_{\mathrm{PI}}}^{\mathrm{CV}+}\left(x_{j}\right)=\left[L_{(r_{\mathrm{low}}),j},U_{(r_{\mathrm{up}}),j}\right],$$

where $L_{(r_{\mathrm{low}}),j}$ and $U_{(r_{\mathrm{up}}),j}$ denote the indicated order statistics of the lower and upper candidate sets, respectively.

After interval construction, the test responses were used only to evaluate empirical coverage according to

$$\mathrm{Coverage}=\frac{1}{n_{\mathrm{te}}}\sum_{j=1}^{n_{\mathrm{te}}}I\left(L_{j}\leq y_{j}\leq U_{j}\right).$$

Interval efficiency was summarized using the mean, median, minimum, and maximum values of the interval width $U_{j}-L_{j}$. This procedure provided an internal assessment of predictive uncertainty and was not interpreted as evidence of external validity or reliable extrapolation beyond the investigated experimental domain.

After the validation procedures and model configuration were finalized, the MLP pipeline was refitted using the complete training subset $D_{\mathrm{tr}}$. Its final predictive performance was then evaluated once on the previously untouched test subset $D_{\mathrm{te}}$ using Equations (22) and (23). All data partitioning, cross-validation, preprocessing, model fitting, and metric calculations were implemented in Python 3.13 using scikit-learn [46].

### 2.7. Explainable Artificial Intelligence (XAI) Analysis

Post hoc explainable artificial intelligence (XAI) methods were applied to improve the interpretability of the fitted MLP model and to quantify how the operational variables contributed to its predictions [35]. Permutation Feature Importance (PFI) was used to evaluate global model reliance on each input variable [51], whereas SHapley Additive exPlanations (SHAP) were used to characterize the local magnitude and direction of the feature contributions [52].

Let $\hat{m}:R^{5}\to R$ denote the fitted MLP predictor and let $D_{\mathrm{te}}={\left\{\left(x_{i},y_{i}\right)\right\}}_{i=1}^{n_{\mathrm{te}}}$ denote the independent test subset, where $y_{i}=C_{t,i}$ is the experimentally measured residual MG concentration. Both XAI analyses were performed after model fitting, without re-estimating the MLP parameters.

Permutation Feature Importance was calculated on the independent test subset using the mean squared error as the evaluation criterion [46,51]. The baseline prediction error of the fitted model was defined as

(27) $$E_{\mathrm{orig}}=\frac{1}{n_{\mathrm{te}}}\sum_{i=1}^{n_{\mathrm{te}}}{\left[y_{i}-\hat{m}\;\left(x_{i}\right)\right]}^{2}.$$

For the *b*-th permutation of feature *j*, the values of that feature were randomly rearranged across the test observations while all other features were kept unchanged. The resulting permuted input vector and prediction error were defined as

(28) $$\begin{array}{cc} x_{i}^{\left(j,b\right)} & ={\left(x_{i1},\ldots ,x_{\pi_{b}\left(i\right),j},\ldots ,x_{iM}\right)}^{T},\\ E_{\mathrm{perm},b}^{\left(j\right)} & =\frac{1}{n_{\mathrm{te}}}\sum_{i=1}^{n_{\mathrm{te}}}{\left[y_{i}-\hat{m}\;\left(x_{i}^{\left(j,b\right)}\right)\right]}^{2}, \end{array}\;j=1,\ldots ,M,$$

where M=5, $\pi_{b}$ denotes the random permutation used in repetition *b*, and *B* denotes the total number of repeated permutations.

The PFI value of feature *j* was calculated as the mean increase in prediction error over the *B* repeated permutations:

(29) $${\bar{FI}}_{j}=\frac{1}{B}\sum_{b=1}^{B}\left(E_{\mathrm{perm},b}^{\left(j\right)}-E_{\mathrm{orig}}\right).$$

The variability of the permutation importance estimate was summarized using

(30) $$s_{FI,j}=\sqrt{\frac{1}{B-1}\sum_{b=1}^{B}{\left[\left(E_{\mathrm{perm},b}^{\left(j\right)}-E_{\mathrm{orig}}\right)-{\bar{FI}}_{j}\right]}^{2}}.$$

A large positive value of ${\bar{FI}}_{j}$ indicates that permuting feature *j* substantially increases the test-set prediction error and, therefore, that the fitted MLP model relies strongly on that feature. Values close to zero indicate limited model reliance, whereas negative values may occur because of sampling variation, redundant information, or interactions among correlated predictors. Accordingly, PFI values were interpreted as model-specific predictive importance rather than as direct causal effects.

To obtain the relative PFI percentages reported in the results, the mean positive permutation importances were normalized as

(31) $$RI_{j}^{\mathrm{PFI}}=100\frac{\max\;\left({\bar{FI}}_{j},0\right)}{\sum_{k=1}^{M}\max\;\left({\bar{FI}}_{k},0\right)}.$$

SHAP analysis was subsequently applied to quantify the contribution of each feature to individual MLP predictions. SHAP represents the prediction for an observation x as an additive decomposition:

(32) $$\hat{m}\left(x\right)=\phi_{0}+\sum_{j=1}^{M}\phi_{j}\left(x\right),$$

where $\phi_{0}$ is the expected model output with respect to the background distribution and $\phi_{j}\left(x\right)$ is the SHAP value assigned to feature *j* for observation x [52].

Let F={1,…,M} denote the complete feature set. The SHAP value of feature *j* was defined as

(33) $$\phi_{j}\left(x\right)=\sum_{S\subseteq F\setminus \{j\}}\frac{|S|!\left(M-|S|-1\right)!}{M!}\left[v_{x}\left(S\cup \{j\}\right)-v_{x}\left(S\right)\right],$$

where *S* is a subset of features not containing *j*, and $v_{x}\left(S\right)$ denotes the expected model prediction when the features in *S* are fixed at their observed values in x, while the residual features are treated according to the background distribution used by the SHAP explainer.

The sign of $\phi_{j}\left(x\right)$ indicates the direction of the feature contribution relative to the baseline prediction $\phi_{0}$. A positive SHAP value increases the predicted residual MG concentration relative to the baseline, whereas a negative SHAP value decreases it. These directional contributions describe the behavior of the fitted model and were not interpreted as causal effects of intervening on the corresponding process variables.

Global SHAP importance was calculated by averaging the absolute local SHAP values over the explained test observations:

(34) $$I_{j}^{\mathrm{SHAP}}=\frac{1}{n_{\mathrm{te}}}\sum_{i=1}^{n_{\mathrm{te}}}\left|\phi_{j}\;\left(x_{i}\right)\right|.$$

The relative SHAP contribution of feature *j* was then calculated as

(35) $$RI_{j}^{\mathrm{SHAP}}=100\frac{I_{j}^{\mathrm{SHAP}}}{\sum_{k=1}^{M}I_{k}^{\mathrm{SHAP}}}.$$

PFI was implemented using the permutation_importance function in scikit-learn, while SHAP values were computed using the Python 3.13 SHAP library [46,52]. The same fitted MLP pipeline and response scale were used for all XAI analyses to ensure consistency between the prediction errors and feature-importance results.

## 3. Results and Discussion

### 3.1. Textural Properties and Adsorption Performance of the SBA-15–Zn–Fe Composite

The available nitrogen adsorption results showed that SBA-15 had a BET specific surface area of $734.79\;m^{2}\;g^{-1}$, a total pore volume of $0.637\;{\mathrm{cm}}^{3}\;g^{-1}$, and a BJH average pore diameter of 3.93nm. For the SBA-15–Zn–Fe composite, the corresponding values were $102.12\;m^{2}\;g^{-1}$, $0.0925\;{\mathrm{cm}}^{3}\;g^{-1}$, and 4.61nm, respectively. Thus, the modification procedure was accompanied by marked reductions in BET specific surface area and total pore volume and an increase in the BJH average pore diameter. Similar changes in textural properties have been reported following metal deposition on SBA-15-based materials [53]. These differences are consistent with substantial modification of the porous texture; however, the nitrogen adsorption results alone do not establish whether the Zn- and Fe-containing species were located inside the pores, deposited on the external surface, or associated with structural alteration. They also do not independently quantify the Zn and Fe contents or conclusively verify retention of the ordered hexagonal SBA-15 structure.

**Figure 1:**
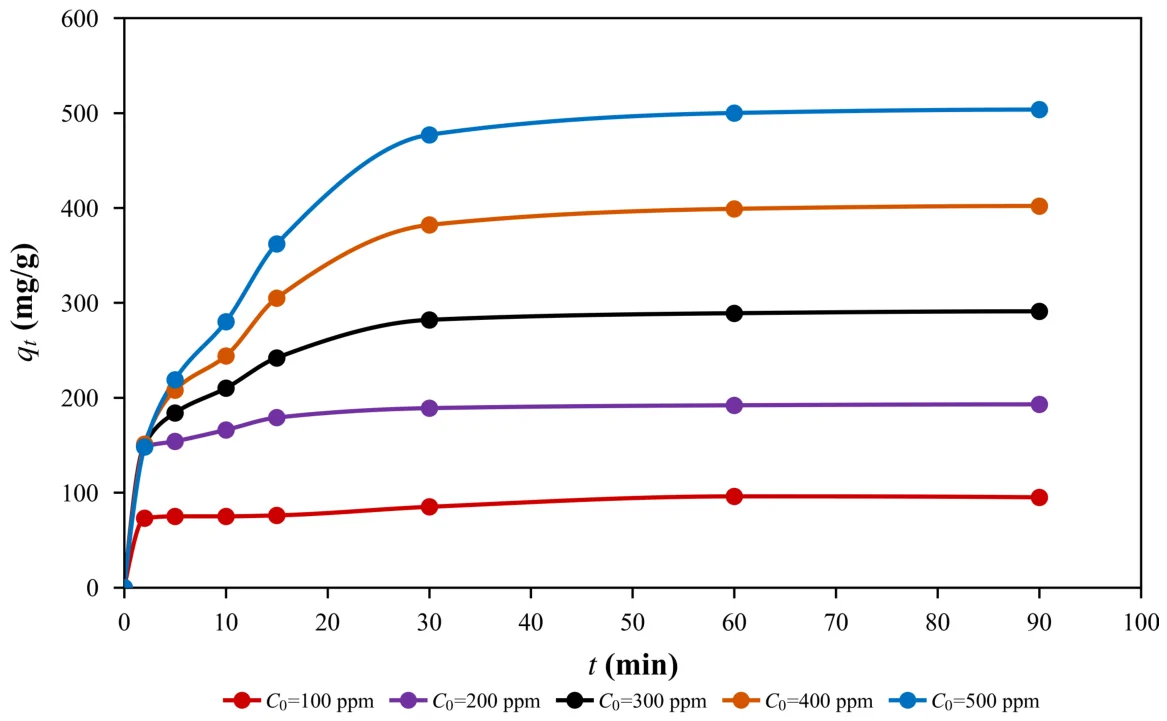
Effect of contact time and initial MG concentration on the adsorption capacity, $q_{t}$, of the SBA-15–Zn–Fe composite (pH=9.0, $X_{0}=1.0$ g L^−1^, and T=45 °C). Symbols represent the experimental observations, whereas the curves are included as guides to the eye.

Figure 1 shows the effects of contact time and initial MG concentration on the adsorption capacity of the SBA-15–Zn–Fe composite.

The adsorption profiles exhibited two distinct stages at all investigated initial MG concentrations. Rapid uptake occurred during the first 15 min, followed by a slower progressive stage. Only small changes in adsorption capacity were observed between 60 and 90 min; therefore, 90 min was used as the equilibrium contact time. The initial rapid stage can be associated with the availability of readily accessible adsorption sites and a relatively large concentration gradient between the aqueous and solid phases. The subsequent decrease in the adsorption rate is consistent with the progressive occupation of the available sites and increasing resistance to further mass transfer.

Within the investigated concentration range, the equilibrium adsorption capacity increased substantially as the initial MG concentration increased from 100 to 500 mg L^−1^. At an initial MG concentration of 500 mg L^−1^, an adsorbent concentration of 1.0 g L^−1^, pH 9.0, and 45 °C, a removal efficiency of 99.65% was obtained after 90 min, while the experimental adsorption capacity approached 500 mg g^−1^. The increase in adsorption capacity with increasing initial concentration can be attributed to the greater concentration gradient and the larger amount of MG available relative to the adsorbent mass.

The equilibrium data yielded an estimated Langmuir maximum monolayer adsorption capacity, $Q_{\max}$, of 1428.57 mg g^−1^ at 45 °C. This model-derived quantity should be distinguished from the experimentally observed equilibrium adsorption capacities within the investigated concentration range.

The performance of the SBA-15–Zn–Fe composite was compared with that of ten MG adsorbents reported in the literature, as summarized in Table 1. Its estimated $Q_{\max}$ was numerically higher than the reported capacities of eight of the ten comparison materials but lower than those reported for the Fe–silica aerogel composite and carbon dots/silica nanoaggregates. In addition to its relatively high estimated capacity, the SBA-15–Zn–Fe composite achieved 99.65% removal at a comparatively high initial MG concentration of 500 mg L^−1^ and reached equilibrium within 90 min.

Nevertheless, direct ranking based only on reported adsorption capacities should be interpreted cautiously because the studies differed in initial concentration, adsorbent dosage, pH, temperature, equilibrium time, material properties, and isotherm-fitting procedures [63]. In particular, model-derived maximum capacities are not necessarily equivalent to experimentally observed uptake values. Subject to these limitations, the results indicate that the SBA-15–Zn–Fe composite is a competitive adsorbent for MG removal within the investigated experimental domain. Further evaluation of regeneration, long-term stability, and performance in real wastewater matrices would be required to establish its practical applicability.

### 3.2. Statistical Analysis

**Figure 2:**
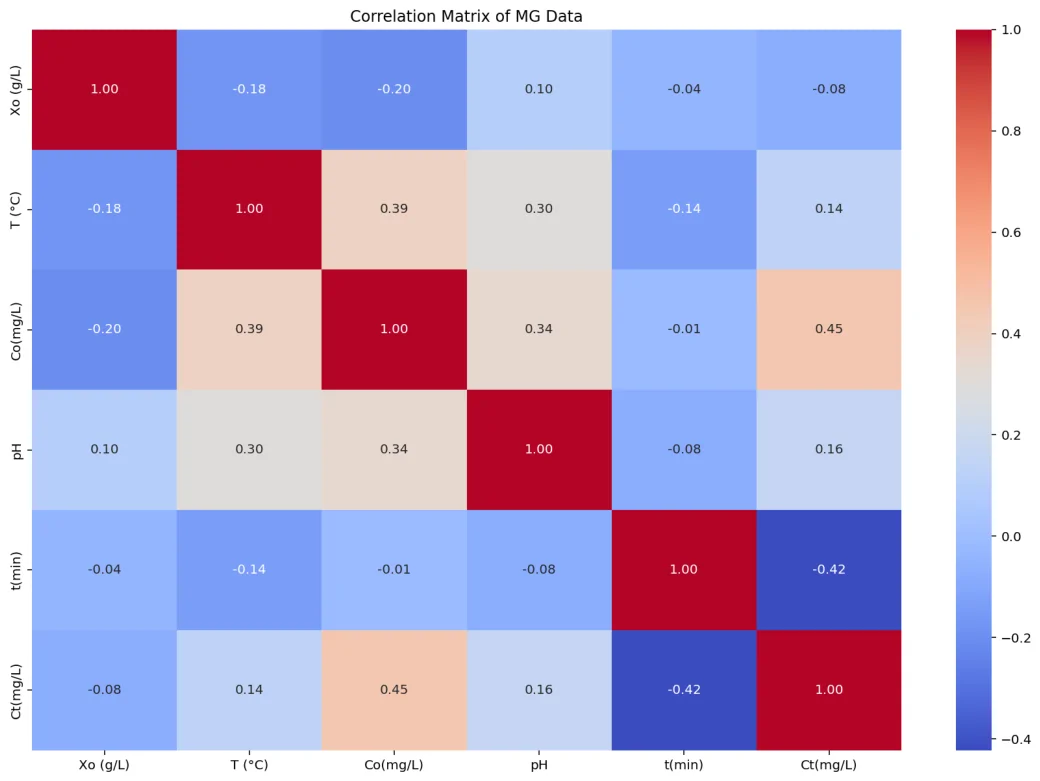
Correlation matrix of the MG adsorption dataset.

Pearson correlation analysis was used to examine the pairwise linear associations between the five operational variables ($X_{0}$, *T*, $C_{0}$, pH, and *t*) and the residual MG concentration ($C_{t}$). The resulting correlation matrix is presented in Figure 2. The correlation coefficients were interpreted as descriptive measures of linear association and not as evidence of causal relationships.

The strongest positive pairwise association with $C_{t}$ was observed for the initial MG concentration ($C_{0}$, r=0.45), whereas contact time exhibited the strongest negative association (*t*, r=−0.42). These results indicate that higher initial MG concentrations were generally associated with higher residual concentrations, while longer contact times were associated with lower residual concentrations. Adsorbent concentration showed only a weak negative association with $C_{t}$ ($X_{0}$, r=−0.08), whereas temperature (*T*, r=0.14) and initial solution pH (r=0.16) showed weak positive pairwise associations. Because these coefficients represent unadjusted bivariate relationships, they do not account for covariation among the operational variables and should not be interpreted as direct or causal effects.

Table 2 summarizes the mean, standard deviation, minimum, and maximum values of the five input variables ($X_{0}$, *T*, $C_{0}$, pH, and *t*) and the response variable ($C_{t}$) for the 315 experimental observations. The observed ranges are consistent with the experimental conditions described in Section 2.3 and characterize the domain used for model development. These descriptive statistics do not establish the representativeness of the dataset beyond the investigated experimental domain.

A multiple linear regression (MLR) model was fitted using ordinary least squares to evaluate the adjusted linear associations between the five input variables and $C_{t}$. The estimated regression coefficients and their corresponding statistical results are presented in Table 3. The model yielded an $R^{2}$ value of 0.389, indicating that the additive linear specification explained 38.9% of the observed variation in $C_{t}$. Thus, a substantial proportion of the response variability remained unexplained by the linear model.

Initial MG concentration and contact time had the largest absolute *t*-statistics in the fitted MLR model. Initial MG concentration was positively associated with $C_{t}$ (β=0.2921, t=9.560, p<0.001), whereas contact time was negatively associated with $C_{t}$ (β=−0.3849, t=−9.607, p<0.001). Temperature also exhibited a statistically significant but smaller adjusted association (β=−1.3373, t=−2.272, p=0.024). In contrast, the coefficients of adsorbent concentration (p=0.692) and initial solution pH (p=0.973) were not statistically significant within the fitted linear model.

The negative adjusted temperature coefficient differs in sign from the weak positive pairwise correlation reported above. This difference arises because the MLR coefficient represents the association between temperature and $C_{t}$ after adjustment for the remaining predictors, whereas the Pearson coefficient represents an unadjusted bivariate association. Accordingly, neither result should be interpreted as an independent causal effect of temperature. Together with the limited $R^{2}$ of the MLR model, these findings indicate that an additive linear specification does not fully represent the relationships contained in the experimental dataset, motivating the subsequent evaluation of a nonlinear predictive model.

The overall MLR model was statistically significant, F(5,309)=39.390 and $p=2.956\times 10^{-31}$, indicating that the five predictors jointly provided greater explanatory ability than an intercept-only model. The term-wise partial *F*-tests are presented in Table 4. Because each predictor had one degree of freedom, these partial *F*-statistics are equivalent to the squared *t*-statistics reported in Table 3 and therefore provide the same variable-level significance conclusions.

Taken together, the correlation and MLR results identified initial MG concentration and contact time as the variables with the strongest linear associations with $C_{t}$. However, the MLR model explained only 38.9% of the observed response variability, indicating that the additive linear specification was insufficient to represent the full structure of the experimental dataset. This result motivates the evaluation of a nonlinear predictive model but does not, by itself, establish the superiority of any particular machine-learning algorithm. The statistical associations identified in this section are subsequently compared with the model-specific PFI and SHAP results.

### 3.3. Evaluation of the Multilayer Perceptron Model

**Figure 3:**
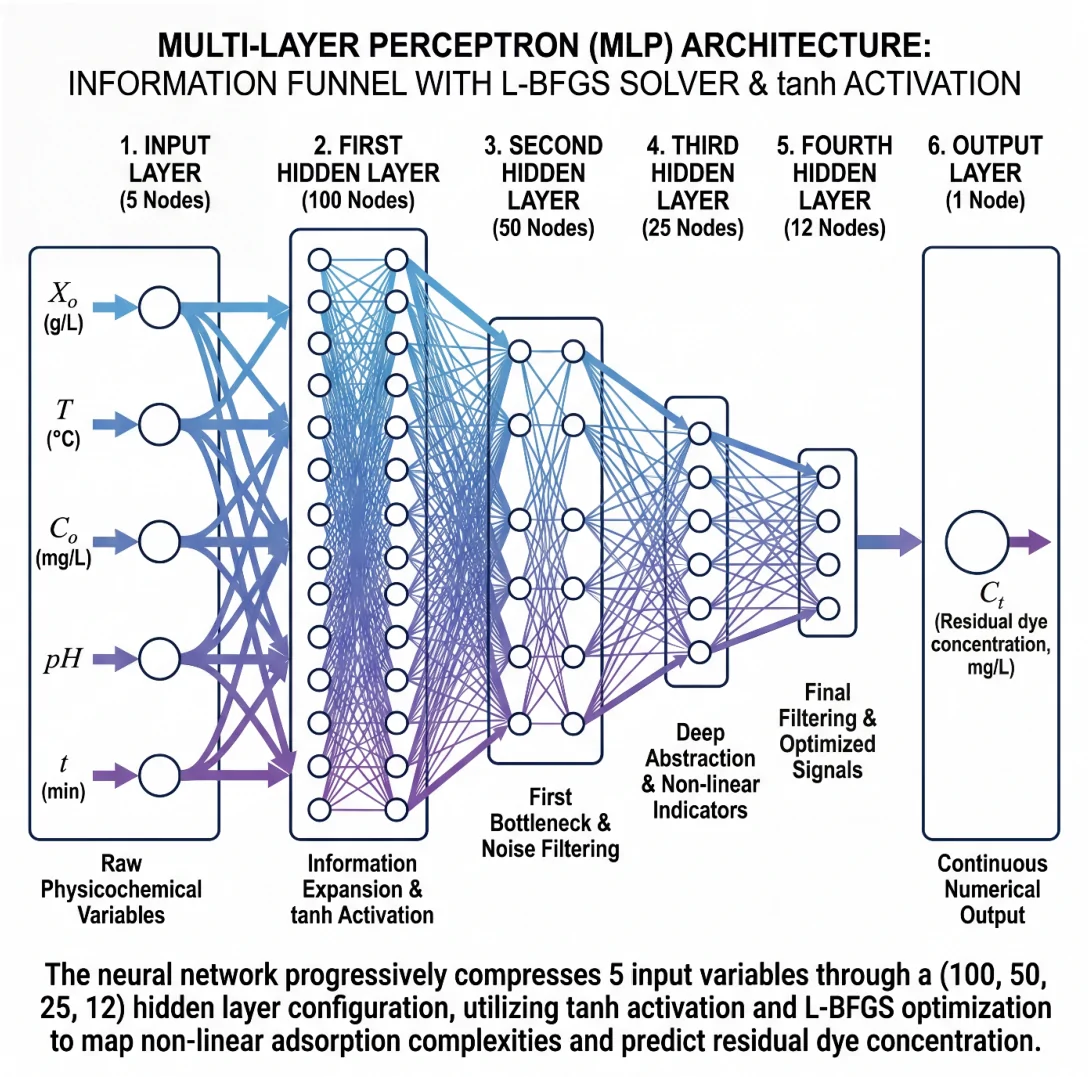
Architecture of the prespecified MLP model, comprising five standardized input variables, four tanh-activated hidden layers with 100, 50, 25, and 12 neurons, and a single continuous output node. Model parameters were estimated using the L-BFGS algorithm.

The prespecified MLP contained five input nodes, four hidden layers with 100, 50, 25, and 12 neurons, and a single regression output node, as illustrated in Figure 3. The first hidden layer expanded the five standardized input variables ($X_{0}$, *T*, $C_{0}$, pH, and *t*) into a 100-dimensional nonlinear representation, after which the layer widths were progressively reduced to 50, 25, and 12 neurons. The topology is therefore described as an expansion–tapering architecture rather than as a conventional bottleneck or information funnel. The hyperbolic tangent activation function was used in all hidden layers [64], and the model parameters were estimated using the full-batch L-BFGS algorithm [45], as detailed in Section 2.5.

Model evaluation followed the protocol described in Section 2.6. The dataset was divided into a development subset (n=252, 80%) and a strictly held-out internal test subset (n=63, 20%). All scaling parameters were estimated exclusively from the corresponding fitting partition and then applied to the validation or test observations without re-estimation, reducing preprocessing-related information leakage [47].

Five-fold cross-validation was used to assess performance variability within the development subset, whereas contact-time-based LOGO-CV evaluated transferability to time groups excluded from model fitting [65]. LOGO-CV was summarized using both group-wise and pooled out-of-fold metrics. Because all excluded contact times remained within the investigated range, LOGO-CV was not interpreted as validation of extrapolation beyond the experimental domain. The held-out test and cross-validation results are reported in Table 5. MSE values were calculated on the standardized response scale, while predictions used for graphical presentation were transformed back to $\mathrm{mg}\;L^{-1}$.

As shown in Table 5, five-fold cross-validation of the prespecified MLP yielded a mean $R^{2}$ of 0.8420 with a fold-wise standard deviation of 0.1273 and a mean standardized-scale MSE of 0.2192. The observed variability across the five folds indicates that predictive performance depended to some extent on the composition of the corresponding fitting and validation partitions.

The contact-time-based LOGO-CV procedure produced a group-wise mean $R^{2}$ of 0.7038 with a standard deviation of 0.2430 and a mean standardized-scale MSE of 0.4030. Because some held-out contact-time groups contained relatively few observations or limited within-group response variability, their individual $R^{2}$ values were unstable. The pooled out-of-fold LOGO-CV result was therefore also calculated, yielding an $R^{2}$ of 0.7411 and a standardized-scale MSE of 0.2589. The pooled result is used as the primary summary of the model’s time-group transferability, whereas the group-wise statistics describe the variability among the individual held-out time groups.

Both LOGO-CV summaries were lower than the five-fold cross-validation result, indicating that prediction was more difficult when an entire contact-time group was excluded from model fitting. However, all held-out contact-time values remained within the experimentally investigated range of 0–360 min. Consequently, LOGO-CV should be interpreted as a structured assessment of time-group transferability within the investigated domain rather than as evidence of reliable extrapolation beyond that domain.

The final MLP, fitted using the complete training subset, achieved an $R^{2}$ of 0.9624 and a standardized-scale MSE of 0.0449 on the strictly held-out internal test subset. This test result demonstrates strong predictive performance for the particular fixed partition. Nevertheless, its higher performance relative to the mean five-fold cross-validation result should not be interpreted as evidence of uniform performance across all possible data partitions. Accordingly, the cross-validation, LOGO-CV, and independent test results provide complementary rather than interchangeable assessments of predictive performance.

**Figure 4:**
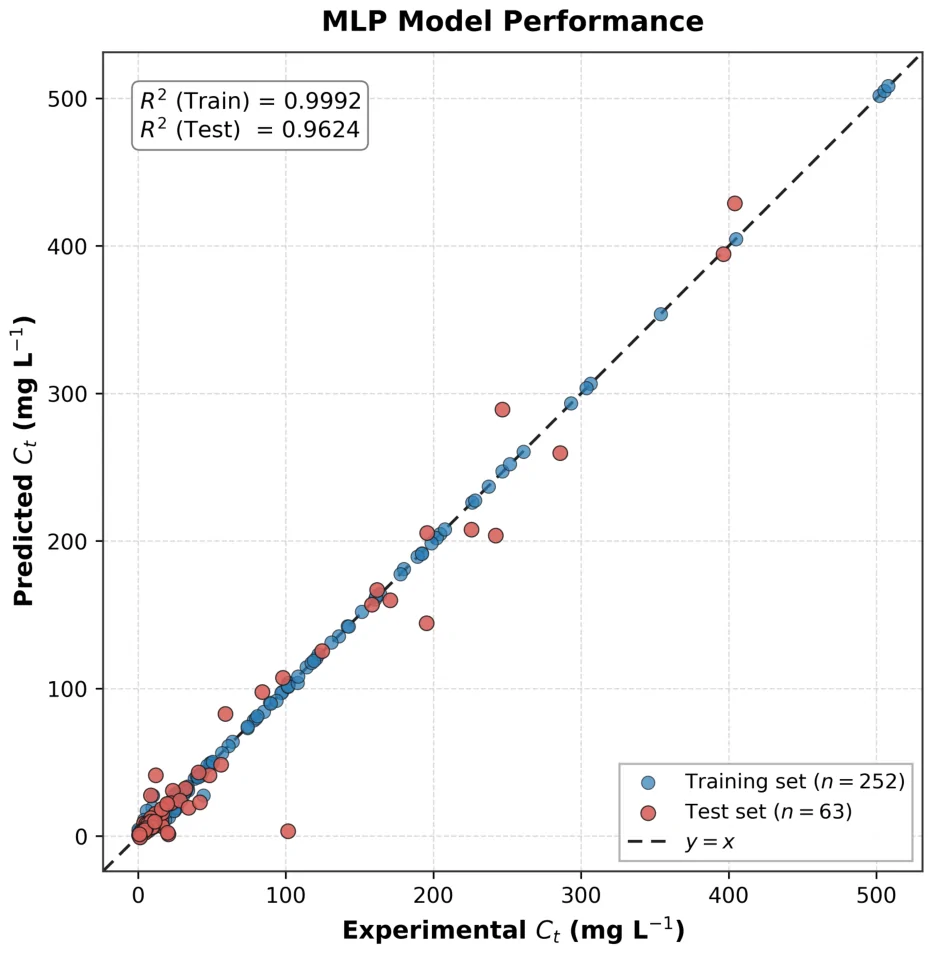
Scatter plot of the experimentally measured and MLP-predicted $C_{t}$ values for the training (n=252) and strictly held-out internal test (n=63) subsets. Predictions were transformed back to the original concentration scale ($\mathrm{mg}\;L^{-1}$) before graphical presentation.

Figure 4 provides an observation-level comparison between the experimentally measured and MLP-predicted $C_{t}$ values. Most training observations were located close to the y=x reference line, whereas the held-out test observations showed several larger deviations, particularly within the intermediate and higher response ranges. The training–test difference indicates that prediction accuracy was not uniform across the complete response range. Accordingly, Figure 4 should be interpreted as a graphical assessment of the fixed training–test partition that complements, but does not replace, the cross-validation and LOGO-CV results summarized in Table 5.

For contextual algorithmic benchmarking, Random Forest regression, standard Gradient Boosting regression, and Support Vector Regression were evaluated using the same fixed training–test partition. As reported in Supplementary Table S1, Random Forest and Gradient Boosting produced higher test-set $R^{2}$ values of 0.9872 and 0.9804, respectively, whereas SVR yielded a substantially lower value of 0.6171. Thus, the MLP was competitive but was not the numerically best-performing model on this partition. The complete model configurations and standardized-scale MSE results are provided in Supplementary Table S1. Because the alternative models were evaluated using the stated configurations without cross-validation-based hyperparameter optimization and the comparison relied on a single test partition, these findings provide a contextual benchmark rather than a definitive ranking of the algorithms. The MLP was retained as the prespecified focal model for evaluating the combined use of group-aware validation, PFI, and SHAP, without implying universal superiority over the alternative models.

The dataset comprised 315 observations, whereas the four-hidden-layer MLP contained 7250 trainable weights and biases; consequently, the risk of overfitting cannot be excluded solely on the basis of the fixed test-set result. Five-fold cross-validation yielded a mean $R^{2}$ of 0.8420 with a fold-wise standard deviation of 0.1273, indicating sensitivity to the composition of the fitting and validation partitions. Under contact-time-based LOGO-CV, the pooled out-of-fold $R^{2}$ decreased to 0.7411, demonstrating reduced transferability when entire contact-time groups were excluded from model fitting. Because all held-out contact-time values remained within the experimentally investigated range, LOGO-CV represents a structured assessment of time-group transferability rather than validation of extrapolation beyond that range. These results do not establish the absence of overfitting or broad external generalizability. Independent validation using newly generated experimental data is therefore required before applying the model to external adsorption systems or operational conditions outside the investigated domain.

The five-fold CV+ analysis produced nominal 95% prediction intervals that contained 62 of the 63 responses in the strictly held-out internal test subset, corresponding to an empirical coverage of 98.41%. On the original concentration scale, the mean and median interval widths were 119.48 and $108.04\;\mathrm{mg}\;L^{-1}$, respectively, while the widths ranged from 103.79 to $264.61\;\mathrm{mg}\;L^{-1}$. Although the observed coverage exceeded the nominal level for this particular test subset, the relatively broad and variable interval widths indicate that predictive uncertainty was not negligible or uniform across the investigated response range. Because the CV+ bounds were constructed without imposing a non-negativity constraint, some lower limits extended below zero; these values represent unconstrained statistical bounds and should not be interpreted as physically possible concentrations. The observation-level prediction intervals are presented in Supplementary Figure S1. These results provide an internal assessment of predictive uncertainty and do not establish coverage validity for external adsorption systems or conditions outside the investigated experimental domain.

### 3.4. Explainable Artificial Intelligence Analysis: PFI and SHAP Results

**Figure 5:**
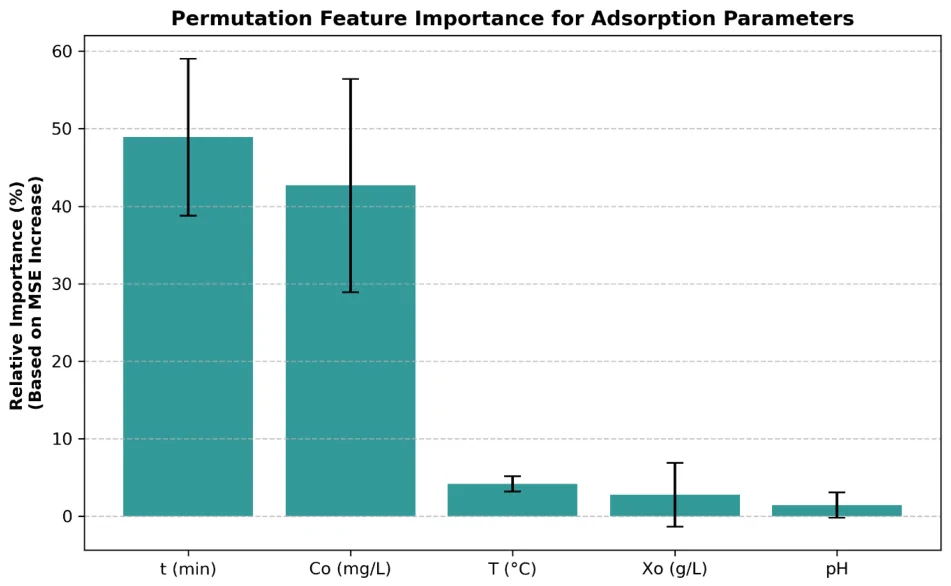
Permutation feature importance values of adsorption parameters.

Despite the high prediction ability of ANN-based models, one of their main limitations is their “black-box” nature, which makes interpreting the nonlinear relationships between input parameters and outputs difficult. In this study, PFI and SHAP algorithms were used together to improve the interpretability of the developed MLP architecture and to evaluate the relationship between model predictions, adsorption behavior, and statistical findings. In the first stage, PFI analysis was applied to assess the global significance of each independent variable by measuring the increase in the MSE after randomly shuffling of the relevant input parameter in the test dataset [51]. The PFI results are presented in Figure 5.

As shown in Figure 5, contact time (*t*, 48.91%) and initial MG concentration ($C_{0}$, 42.67%) were identified as the two variables with the largest effects on the predictive performance of the fitted MLP. Their combined normalized PFI contribution of 91.58% indicates that the model relied predominantly on time- and concentration-dependent information when predicting residual MG concentration.

This result is consistent with the experimentally observed adsorption profiles, which exhibited rapid initial uptake followed by a slower approach to equilibrium. The importance of contact time may therefore reflect the progressive occupation of accessible adsorption sites and the increasing resistance to further mass transfer as adsorption proceeds. External mass transfer and transport within the porous adsorbent structure may both contribute to this time dependence. However, PFI does not distinguish between these transport processes or identify a unique rate-controlling step; such a conclusion would require dedicated diffusion-model diagnostics.

The importance of initial MG concentration is consistent with the concentration gradient between the liquid phase and the adsorbent surface, which provides a driving force for the mass transfer of dye molecules toward the adsorbent structure [66,67,68]. Higher initial concentrations also increase the amount of MG available relative to the adsorbent mass and may promote progressive occupation of the available adsorption sites. Thus, the PFI results agree with the observed kinetic and concentration-dependent behavior, but should not be interpreted as direct causal evidence for a specific adsorption mechanism.

**Figure 6:**
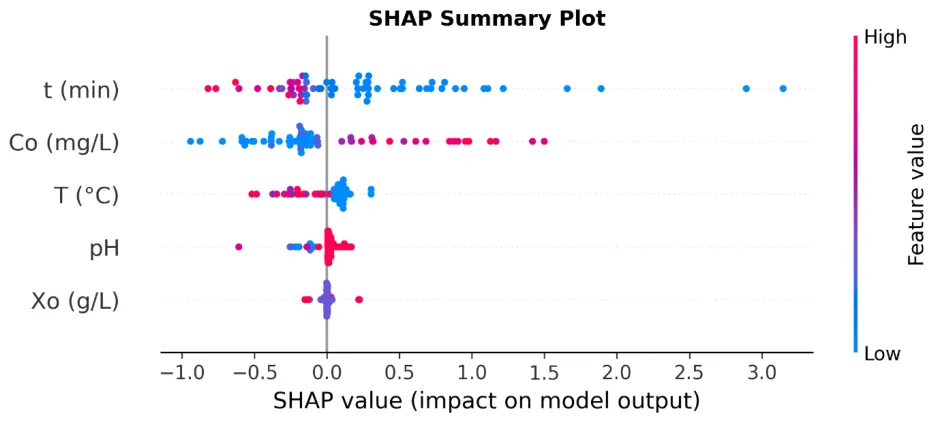
SHAP summary plot and parameter effects calculated for the MLP model.

The SHAP summary plot to evaluate the directional effects of features and their marginal contributions to individual predictions based on a game theory approach is presented in Figure 6. In the SHAP summary plot, the color scale represents the magnitude of the corresponding feature value (red: high value, blue: low value), while the horizontal axis (SHAP value) denotes the contribution of each parameter to the model output, corresponding to the residual dye concentration ($C_{t}$) in the liquid phase [69]. As shown in Figure 6, SHAP analysis demonstrated a feature importance trend ($t>C_{0}>T>\mathrm{pH}>X_{0}$) that is consistent with the hierarchy obtained from PFI analysis and also provided information about the directional effects of each parameter.

For t, the distribution of high feature values (red points) on the negative side of the SHAP axis indicates that increasing adsorption time contributes to a decrease in the predicted $C_{t}$ values. This behavior is consistent with the time-dependent decrease in residual dye concentration during the adsorption process. In contrast, observations with high initial MG concentrations ($C_{0}$, red points) are mostly located in the positive SHAP region and this suggests that increasing the initial dye concentration leads to higher residual dye concentrations due to the increased amount of dye entering the system and approaching adsorbent saturation.

The most notable difference between the PFI and SHAP results was observed for temperature (*T*). Temperature accounted for 4.20% of the normalized PFI contribution, whereas its relative mean absolute SHAP contribution was 12.02%. Because PFI and SHAP quantify different aspects of model behavior, these percentages should not be interpreted as directly equivalent measures of physical influence.

In the SHAP summary plot, higher temperature values were predominantly associated with negative SHAP values, indicating that the fitted MLP generally associated increasing temperature with lower predicted residual MG concentrations within the investigated range of 25–45 °C. This model-derived trend is physicochemically consistent with reduced solution viscosity, increased molecular mobility, and facilitated transport of MG molecules toward and within the porous adsorbent structure [70,71,72,73,74,75].

Nevertheless, SHAP values represent directional contributions to model predictions rather than causal or thermodynamic evidence. Therefore, the observed temperature dependence does not independently establish an endothermic adsorption mechanism or demonstrate an increase in the affinity of the Zn/Fe-modified surface sites for MG. Such conclusions would require supporting thermodynamic parameters, particularly $\Delta H^{\circ }$, together with independent equilibrium and surface characterization. The present results support a temperature-facilitated adsorption trend within the investigated experimental domain but do not identify a unique thermodynamic mechanism. In contrast, pH and adsorbent concentration ($X_{0}$) remained at lower importance levels in both analyses, indicating that the fitted model relied less strongly on these variables within the examined experimental ranges.

The proposed XAI-assisted MLP and conventional adsorption modeling approaches serve related but distinct purposes. Adsorption isotherm and kinetic models provide parsimonious mathematical descriptions and fitted quantities, such as model-dependent maximum capacities and apparent rate constants, that can support physicochemical interpretation under the assumptions of the selected model [76,77]. However, the validity of these interpretations depends on the adequacy of the prescribed model structure, and goodness of fit alone does not establish a unique adsorption mechanism. Response surface methodology can represent main effects, quadratic terms, and pairwise interactions and can support optimization within a designed experimental region [78]. Nevertheless, a conventional second-order response surface remains a local polynomial approximation whose adequacy depends on the experimental design and investigated factor space.

In contrast, the MLP provides a flexible multivariable mapping from $X_{0}$, *T*, $C_{0}$, pH, and *t* to $C_{t}$ without imposing a predefined equilibrium, kinetic, or quadratic response equation. This flexibility is accompanied by greater dependence on the amount and representativeness of the training data, reduced parameter-level interpretability, and limited extrapolation beyond the sampled domain. PFI and SHAP partially improve model transparency by describing global model reliance and local feature contributions, respectively; however, they do not yield equilibrium, kinetic, or thermodynamic constants and should not be interpreted as direct evidence of causality or adsorption mechanism. The conventional and data-driven approaches should therefore be regarded as complementary rather than interchangeable. From an engineering perspective, the developed MLP should be interpreted as a surrogate modeling component rather than as a standalone optimization or process-control system. Within the experimentally investigated domain, the model can rapidly screen candidate combinations of initial MG concentration, adsorbent concentration, pH, temperature, and contact time by estimating the corresponding residual MG concentration. In a future decision-support framework, the fitted model could be coupled with a constrained optimization algorithm to identify operating conditions that satisfy a specified effluent-concentration limit while accounting for objectives such as adsorbent consumption, treatment time, and temperature-related energy demand. Following appropriate external validation, the upper CV+ prediction limit could also be used as a conservative compliance criterion instead of relying solely on the point prediction. However, the present study did not implement a formal optimization problem, define an industrial cost function, or validate process-control performance. The proposed implementation should therefore be regarded as a prospective workflow rather than a demonstrated industrial application.

Although the developed MLP model showed strong predictive performance for MG adsorption onto the SBA-15–Zn–Fe composite on the fixed internal test partition, its applicability should be interpreted strictly within the experimental limits of the present study. The model was trained using observations generated within the investigated ranges of contact time (0–360 min), initial MG concentration (100–500 mg L^−1^), adsorbent concentration (0.5–3.0 g L^−1^), pH (5–9), and temperature (25–45 °C). The pooled out-of-fold LOGO-CV performance ($R^{2}=0.7411$) was lower than the mean five-fold cross-validation performance ($R^{2}=0.8420$). Moreover, the group-wise LOGO-CV results showed substantial variability ($R^{2}=0.7038\pm 0.2430$) across the 15 held-out contact-time groups. These findings indicate reduced transferability when entire contact-time groups are excluded from model fitting. However, because all held-out contact-time values remained within the experimentally observed range, LOGO-CV does not establish the model’s ability to extrapolate beyond the investigated experimental domain.

Accordingly, predictions outside the specified operational ranges have not been validated and should not be relied upon without additional experimental evidence, model retraining, and independent external validation. Furthermore, the purely data-driven MLP does not impose mechanistic constraints that would guarantee physically consistent predictions outside its training domain. Because the dataset was obtained from batch adsorption experiments conducted under controlled laboratory conditions using synthetic aqueous solutions, the model may not fully represent the behavior of the adsorption system in real wastewater matrices, continuous-flow configurations, newly synthesized composite batches, or other experimental settings. Future research should therefore evaluate the model using independently generated datasets that extend the investigated operational ranges and include real industrial effluents and pilot-scale or continuous-flow adsorption systems. Application to different adsorbents, dye types, or wastewater matrices would also require model retraining and independent validation.

A further limitation is that the available physicochemical characterization was restricted to nitrogen adsorption-derived textural parameters. Low-angle X-ray diffraction or transmission electron microscopy data were not available to independently confirm retention of the ordered SBA-15 mesostructure, and elemental analyses such as ICP-OES, XRF, or SEM-EDS were not available to quantify the actual Zn and Fe contents. Consequently, the SBA-15–Zn–Fe designation reflects the stated synthesis and modification procedure, whereas the intended metal composition and the location of the metal-containing species were not independently verified in the present study.

## 4. Conclusions

This study evaluated MG adsorption by the material prepared through the stated SBA-15–Zn–Fe synthesis procedure and developed an XAI-assisted MLP framework for predicting residual MG concentration. Under the investigated conditions, a removal efficiency of 99.65% was obtained at an initial MG concentration of 500 mg L^−1^, an adsorbent concentration of 1.0 g L^−1^, pH 9.0, 45 °C, and a contact time of 90 min. The Langmuir-estimated maximum monolayer capacity was 1428.57 mg g^−1^; this model-derived value should not be interpreted as an experimentally observed uptake at 500 mg L^−1^.

The prespecified MLP achieved an $R^{2}$ of 0.9624 on the strictly held-out internal test subset. However, its lower pooled LOGO-CV performance ($R^{2}=0.7411$) showed that prediction became more difficult when entire contact-time groups were excluded from model fitting. The CV+ uncertainty analysis provided 98.41% empirical coverage for nominal 95% prediction intervals, but the relatively large and variable interval widths indicated that predictive uncertainty was not negligible. In addition, Random Forest and Gradient Boosting achieved higher test-set accuracy than the MLP in the contextual single-partition comparison. The MLP results therefore do not establish universal predictive superiority over the alternative algorithms.

PFI and SHAP consistently identified contact time and initial MG concentration as the variables on which the fitted MLP relied most strongly. SHAP indicated that longer contact times were generally associated with lower predicted residual MG concentrations, whereas higher initial concentrations were associated with higher predicted residual concentrations. Temperature had a secondary contribution. These model-specific associations were consistent with the observed adsorption trends but should not be interpreted as independent causal, kinetic, or thermodynamic evidence.

The proposed framework should be used only for interpolation within the investigated ranges of contact time (0–360 min), initial MG concentration (100–500 mg L^−1^), adsorbent concentration (0.5–3.0 g L^−1^), pH (5–9), and temperature (25–45 °C). Its applicability to real wastewater, continuous-flow systems, newly synthesized material batches, and conditions outside these ranges has not been established. Moreover, the available physicochemical characterization was limited to nitrogen-adsorption-derived textural parameters and did not independently verify the ordered SBA-15 structure or quantify the Zn and Fe contents. External experimental validation and additional structural and elemental characterization are therefore required before practical process optimization or industrial implementation. 

## Figures and Tables

**Table 1:** Reported operating conditions and adsorption performance of silica-based and other adsorbents used for MG removal.

Adsorbent	Operating Conditions					Adsorption Performance		Reference
	pH	$C_{0}$ (mg L^−1^)	$X_{0}$ (g L^−1^)	Time (min)	T (°C)	Reported Capacity (mg g^−1^)	Removal Efficiency (%)	
${\mathrm{Eu}}^{3+}$-MOF	7.0	10	2.0	120	35	33.7	97.64	[54]
SiO_2_–MgO composite	8.0	80	0.6	120	25	115.64	97.72	[55]
Fe–silica aerogel composite	7.0	1500	1.0	720	35	1592	>90	[56]
Polymer hybrid bio-nanocomposite	7.0	150	1.0	40	50	384.615	99.79	[57]
Si/Fe–ZnNPs	Natural (≈3.3)	100	1.0	180	50	666.67	96.81	[6]
Ca-ALG@MCM-41	Natural	30	6.6	60	25	142.14	97.62	[58]
Calcium silicate nanopowders	6.5	10	0.7	60	30	59.95	100	[59]
Carboxymethyl cellulose-based silica hydrogel nanocomposite	7.0	100–600	0.66	60	25	906.3	–	[60]
Hydrochar-grafted chitosan	7.5	25	0.4	420	25	420.02	96.47	[61]
Carbon dots/silica nanoaggregates	6.0	200	0.05	2880	35	4091	–	[62]
SBA-15–Zn–Fe composite	9.0	500	1.0	90	45	1428.57	99.65	This study

Note: $C_{0}$, initial MG concentration; $X_{0}$, adsorbent concentration. The value reported for the SBA-15–Zn–Fe composite corresponds to the Langmuir-estimated maximum monolayer adsorption capacity, $Q_{\max}$. The adsorption capacities and removal efficiencies reported for the comparison materials were obtained under different experimental conditions and may not represent directly equivalent performance measures. Consequently, the comparison should be interpreted cautiously and should not be regarded as a strictly standardized ranking.

**Table 2:** Descriptive statistics of independent and dependent variables in the adsorption dataset.

Parameter	$X_{0}$ (g/L)	*T* (°C)	$C_{0}$ (mg/L)	pH	*t* (min)	$C_{t}$ (mg/L)
Mean	1.105	30.397	220.635	8.556	86.111	51.436
Standard deviation	0.426	7.535	147.332	1.067	99.344	88.327
Minimum	0.500	25.000	100.000	5.000	0.000	0.085
Maximum	3.000	45.000	500.000	9.000	360.000	507.917

**Table 3:** Statistical parameters and coefficients of the MLR model ($R^{2}=0.389$).

Variable	Coefficient	Standard Error	*t*-Value	*p*-Value
Constant	63.8661	34.941	1.828	0.069
$X_{0}$ (g L^−1^)	−3.8491	9.700	−0.397	0.692
*T* (°C)	−1.3373	0.589	−2.272	0.024
$C_{0}$ (mg L^−1^)	0.2921	0.031	9.560	<0.001
pH	0.1374	4.086	0.034	0.973
*t* (min)	−0.3849	0.040	−9.607	<0.001

**Table 4:** Term-wise partial *F*-tests for the multiple linear regression model of residual MG concentration, $C_{t}$.

Model Term	Sum of Squares	df	*F*-Statistic	*p*-Value
Adsorbent concentration ($X_{0}$)	762.40	1	0.157	0.692
Temperature (*T*)	24,998.30	1	5.163	0.024
Initial MG concentration ($C_{0}$)	442,522.00	1	91.396	<0.001
Initial solution pH	5.47	1	0.001	0.973
Contact time (*t*)	446,843.00	1	92.289	<0.001
Residual error	1,496,120.00	309	N/A	N/A

Note: Statistical significance was assessed at p<0.05; N/A indicates not applicable.

**Table 5:** Cross-validation and independent test-set performance of the prespecified MLP model. MSE values were calculated on the standardized response scale.

Validation Strategy	$R^{2}$	SD of $R^{2}$	Standardized MSE
Five-fold CV (mean across five folds)	0.8420	0.1273	0.2192
LOGO-CV (group-wise mean)	0.7038	0.2430	0.4030
LOGO-CV (pooled out-of-fold)	0.7411	N/A	0.2589
Independent test set (held-out 20%)	0.9624	N/A	0.0449

Note: Five-fold CV values are the mean results obtained across the five validation folds. The group-wise LOGO-CV values are the unweighted mean and standard deviation across the 15 held-out contact-time groups. The pooled LOGO-CV metrics were calculated by combining the out-of-fold predictions from all held-out contact-time groups. MSE values were calculated on the standardized response scale. N/A: not applicable.

## Data Availability

The experimental data supporting the findings of this study are available from the corresponding author upon reasonable request. The Python 3.13 code used for data analysis, MLP modeling, model validation, and XAI analysis is publicly available at https://github.com/metinzontul/malachite-green-mlp-xai (accessed on 24 August 2026).

## Supplementary Materials

The following supporting information can be downloaded at: https://www.mdpi.com/article/10.3390/molecules31173005/s1, MG3; Table S1: Independent internal test-set performance of the prespecified multilayer perceptron (MLP) and alternative machine-learning models using the fixed 80/20 training–test partition; Table S2: Textural parameters of SBA-15 and the SBA-15–Zn–Fe composite obtained from nitrogen adsorption analysis; Figure S1: Held-out test observations ordered by $C_{t}$.

## Abbreviations

The following abbreviations are used in this manuscript:

ANN	Artificial neural network
ANFIS	Adaptive neuro-fuzzy inference system
L-BFGS	Limited-memory Broyden–Fletcher–Goldfarb–Shanno
LOGO-CV	Leave-one-group-out cross-validation
MG	Malachite green
ML	Machine learning
MLP	Multilayer perceptron
MSE	Mean squared error
PFI	Permutation feature importance
SHAP	SHapley Additive exPlanations
XAI	Explainable artificial intelligence
