# Supplementary material for: Explainable Artificial Intelligence Assisted Modeling of Malachite Green Adsorption onto SBA-15–Zn–Fe Composite
Source: Molecules. 2026 Aug 27;31(17):3005. doi: 10.3390/molecules31173005 (PMC13566982; doi:10.3390/molecules31173005)
Supplement: Supplementary file 1 [file molecules-31-03005-s001.zip › Supplementary_Materials.pdf]

Supplementary Materials

Explainable Artificial Intelligence Assisted Modeling of Malachite Green Adsorption onto SBA-15-Zn-Fe Composite

Memduha Ergüt, Salih Ozbay, Seda Karateke, and Metin Zontul

Supplementary Table S1. Independent internal test-set performance of the prespecified multilayer perceptron (MLP) and alternative machine-learning models using the fixed 80/20 training-test partition.

Model	Model configuration	Test R^2	Standardized-response MSE
Multilayer Perceptron (MLP)	Hidden layers: 100-50-25-12; activation: tanh; solver: L-BFGS; L2 regularization: 0.0001; maximum iterations: 2000; random_state: 42	0.9624	0.0449
Random Forest	Estimators: 100; maximum depth: unrestricted; max_features: 1.0; bootstrap: True; random_state: 42	**0.9872**	**0.0153**
Gradient Boosting	Estimators: 100; learning rate: 0.1; maximum depth: 3; loss: squared error; random_state: 42	0.9804	0.0234
Support Vector Regression (SVR)	Kernel: radial basis function; C: 1.0; epsilon: 0.1; gamma: scale	0.6171	0.4574

Note: The complete dataset (n = 315) was divided once into a training subset (n = 252) and a strictly held-out internal test subset (n = 63) using random_state = 42. Input and response standardization parameters were estimated using the training subset only and then applied to the test subset. MSE values are reported in standardized-response units. The alternative models were evaluated using the scikit-learn configurations shown above, without cross-validation-based hyperparameter optimization. Bold values indicate the best independent test-set performance.

Supplementary Materials

Supplementary Table S2. Textural parameters of SBA-15 and the SBA-15-Zn-Fe composite obtained from nitrogen adsorption analysis.

Material	S_{BET} ($m^2 g^{-1}$)	V_T ($cm^3 g^{-1}$)	d_{BJH} (nm)
SBA-15	734.79	0.6370	3.93
SBA-15-Zn-Fe composite	102.12	0.0925	4.61

Note: S_{BET}, BET specific surface area; V_T, total pore volume; d_{BJH}, BJH average pore diameter. The values were obtained from the available nitrogen adsorption analysis records. These textural parameters do not independently quantify the Zn and Fe contents or confirm retention of the ordered hexagonal SBA-15 structure.

Interpretive scope. Relative to SBA-15, the modified composite showed lower BET specific surface area and total pore volume and a higher BJH average pore diameter. These differences demonstrate a marked alteration of the measured textural properties after the modification procedure. Nitrogen adsorption alone, however, cannot determine whether Zn- and Fe-containing species are located inside the pores, deposited on the external surface, or associated with structural alteration.

Supplementary Materials

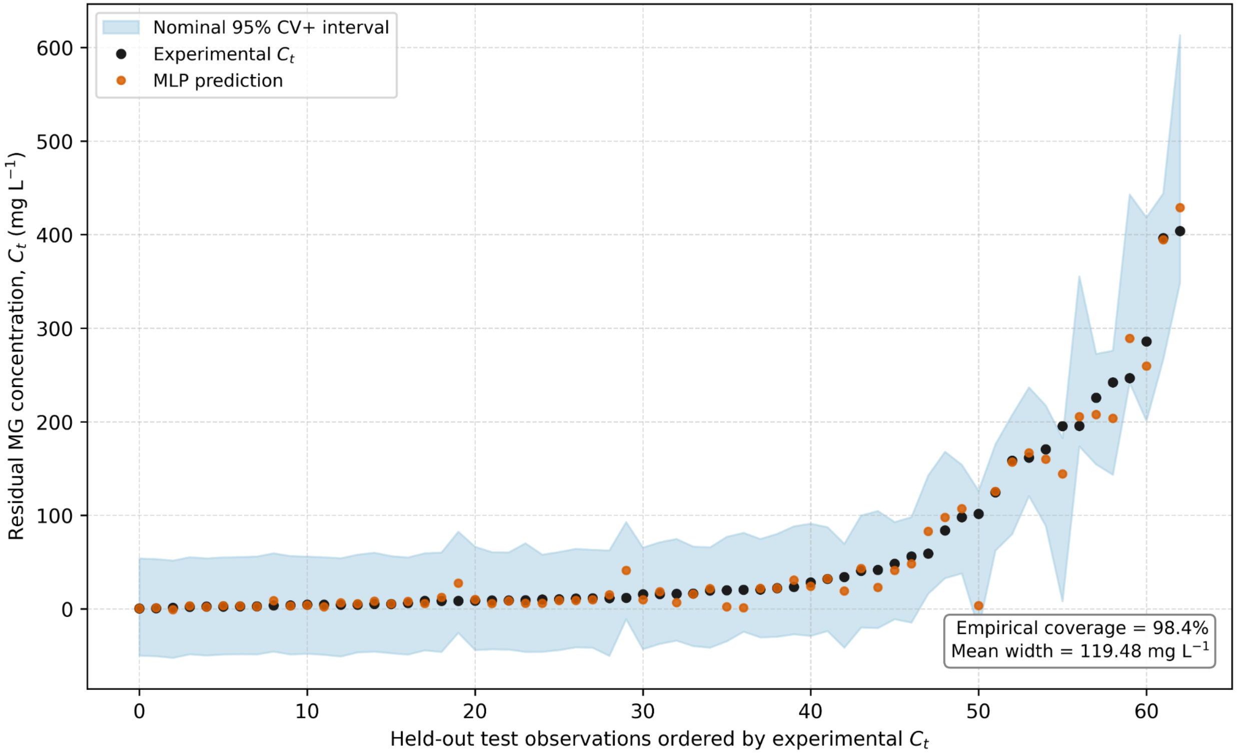

Supplementary Figure S1. Held-out test observations ordered by C_t
